# Viral Infection and Dissemination Through the Lymphatic System

**DOI:** 10.3390/microorganisms13020443

**Published:** 2025-02-18

**Authors:** Morgan E. Brisse, Heather D. Hickman

**Affiliations:** Laboratory of Viral Diseases, National Institute of Allergy and Infectious Diseases, National Institutes of Health, Bethesda, MD 20852, USA; brisseme@nih.gov

**Keywords:** lymph node, lymph-borne virus, sinus macrophage, dendritic cell, antigen presentation, monocyte, NK cell, cytokine, B cell activation, T cell activation

## Abstract

Many viruses induce viremia (virus in the blood) and disseminate throughout the body via the bloodstream from the initial infection site. However, viruses must often pass through the lymphatic system to reach the blood. The lymphatic system comprises a network of vessels distinct from blood vessels, along with interconnected lymph nodes (LNs). The complex network has become increasingly appreciated as a crucial host factor that contributes to both the spread and control of viral infections. Viruses can enter the lymphatics as free virions or along with migratory cells. Once virions arrive in the LN, sinus-resident macrophages remove infectious virus from the lymph. Depending on the virus, macrophages can eliminate infection or propagate the virus. A virus released from an LN is eventually deposited into the blood. This unique pathway highlights LNs as targets for viral infection control and for modulation of antiviral response development. Here, we review the lymphatic system and viruses that disseminate through this network. We discuss infection of the LN, the generation of adaptive antiviral immunity, and current knowledge of protection within the infected node. We conclude by sharing insights from ongoing efforts to optimize lymphatic targeting by vaccines and pharmaceuticals. Understanding the lymphatic system’s role during viral infection enhances our knowledge of antiviral immunity and virus–host interactions and reveals potential targets for next-generation therapies.

## 1. Introduction

The lymphatic system consists of lymphatic vessels and an array of interconnected lymph nodes (LNs). LNs are recognized for their pivotal role in initiating and developing adaptive immunity [1]. Their highly organized structure facilitates optimal interactions between B cells, T cells, and antigen-presenting cells (APCs), which are essential for lymphocyte activation and clonal expansion [2]. Consequently, LNs function as centralized hubs for B and T cell activation, enabling both recruited and resident innate immune cells to assist in and modulate this process. The widespread distribution of LNs throughout the body allows for a swift and effective response to infections occurring in various peripheral tissues [3]. This strategic placement ensures the immune system can rapidly identify and address threats, maintaining the body’s overall health and resilience against disease.

Although LNs are primarily known as the site for initiating an adaptive immune response, our understanding of the role of the entire lymphatic system during the spread of viruses from initial infection sites has recently grown. When a virus infects the skin through insect bites, enters the respiratory tract via inhalation, or is ingested through the digestive tract, local inflammation, infectious virus, and viral antigen (Ag) can all be rapidly relayed to the draining LN. Within the LN, viral infection varies by the tropism of the virus and access to cells permissive to infection. Hence, LN-resident cells efficiently remove viral particles from some viruses from the lymphatic fluid, preventing downstream spread and eventual deposition into the blood. However, other viruses can productively infect LN cells, producing new viruses and then viral movement to downstream LNs and the blood. In some cases, viral amplification in LNs can even be essential for systemic infection. Some historically significant viruses, including smallpox, exhibited intrahost spread via this route, called lymphohematogenous dissemination.

As our ability to target specific cells and tissues continues to improve, LNs are a prime site for expediting immune responses to viral infections and enhancing responses to preventative therapeutics such as vaccines. In this review, we follow the path of a virus through the lymphatic network and discuss immune countermeasures to prevent its spread. We describe the anatomy and physiology of the lymphatic system, known viral infections of the LN, the mechanisms of lymph-borne viral spread, and the contributions of LN-resident and circulating immune cells to controlling LN infection. We also describe ongoing efforts to improve the therapeutic targeting of LNs, with lessons that can be applied to our understanding of viral movement in the lymphatics. Furthering our knowledge of lymphatic-borne viruses is essential in developing the next generation of antiviral vaccines and therapeutics.

## 2. Anatomy of the Lymphatic System

### 2.1. Lymph Circulation Through Lymphatic Vessels

**Figure 1:**
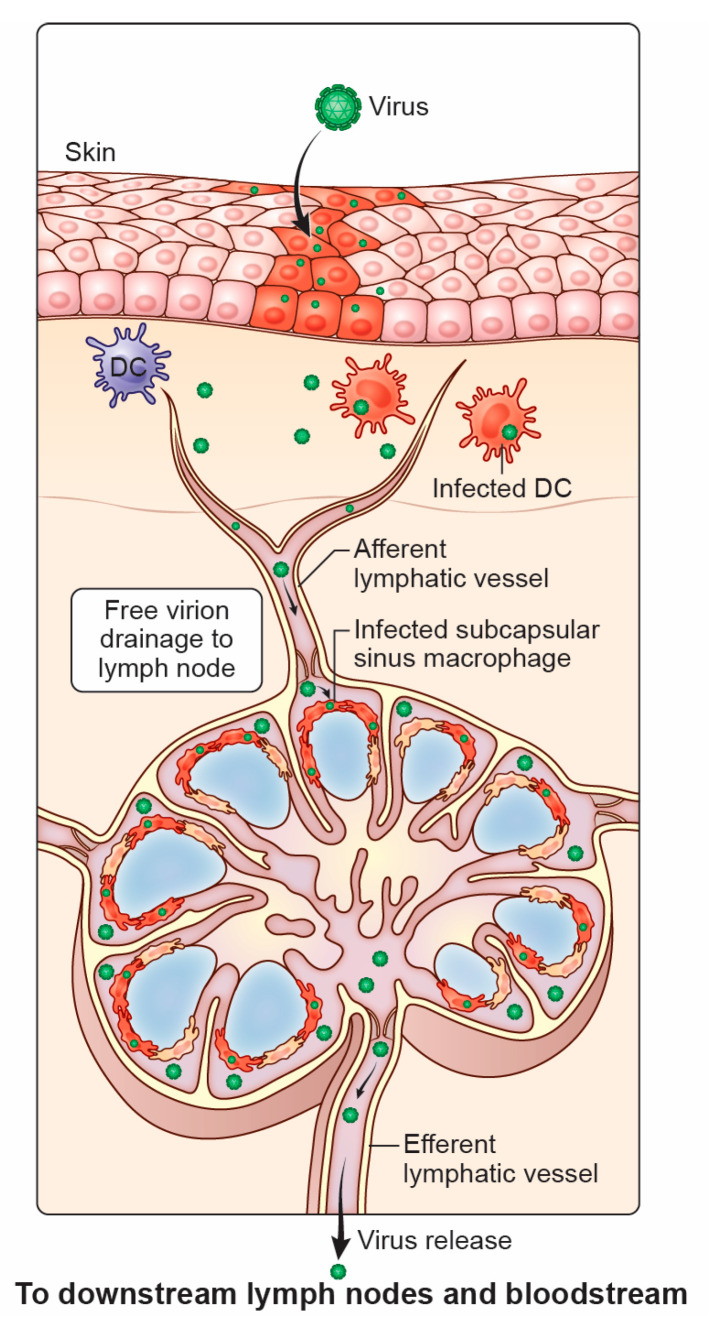
The lymphatic system captures viruses that drain from the tissue. Free virions, or infected or viral antigen-carrying migratory cells (such as dendritic cells), enter lymphatic vessels near the initial site of infection (such as the skin). Lymph fluid unidirectionally flows through lymphatic vessels, eventually flowing into lymph node sinuses lined by lymphatic endothelial cells (LECs). Incoming virions can be captured by subcapsular sinus macrophages (SSMs) and medullary sinus macrophages (MSMs). Any uncaptured virus flows out of the lymph node through the efferent lymphatic vessel.

The lymphatic system is comprised of a circulatory network of lymphatic vessels connected to LNs [4,5] (Figure 1). Lymph fluid first enters small lymphatic vessels in the tissue as free fluid primarily filtered from blood capillaries, driven by hydrostatic pressure within the blood vessels [4,5]. Lymph begins its transit through the lymphatic system via the initial lymphatic vessels, the most terminal and smallest vessels (ranging in size from 10 to 60 µm [6] diameter). Initial lymphatic vessels are formed by a single layer of lymphatic endothelial cells (LECs) interconnected by button-like junctions [7] and do not contain a basement membrane or perivascular cells such as pericytes or smooth muscle cells [8]. Thus, fluid can enter initial lymphatic vessels between the button-like junctions without the impedance of a basement membrane. The initial lymphatics merge into larger collecting lymphatics (50–200 µm diameter) containing a basement membrane, smooth muscle, and pericytes. Instead of connecting through button-like junctions, LECs in collecting lymphatics possess tight, zipper-like junctions. Collecting lymphatics also contains LEC-lined valves within the vessel lumen that prevent backflow [6].

Several factors drive entry into and circulation through lymphatic vessels. Interstitial pressure (i.e., the pressure within the space between cells in the tissue) pushes fluid into the initial lymphatics. The lymphatic vessels also respond to increases in interstitial pressure during an inflammatory event in peripheral tissues by opening valves between LEC-button junctions to promote fluid intake [9]. For lymphatic vessels above the heart and thoracic duct, gravity also enhances lymph entry into lymphatic vessels. Accordingly, anatomically lower lymphatic vessels, including those in the legs, must overcome the force of gravity to move fluid uphill [10]. Specialized regions of collecting lymphatic vessels between one-way valves, termed lymphangions, use muscle to contract the vessels and regularly pump lymph forward against hydrostatic pressure gradients and gravity [11].

Fluid flow into lymphatic vessels is also driven by suction created by lymphatic pumping and high interstitial pressure in LNs generated by dense architectural cellular networks, including stromal cells and blood vessels, under internal tensile pressure even under naïve conditions [12,13,14,15,16,17]. The pressure inside LNs further increases during an immune reaction or inflammatory stimulation [18]. The rate of lymph fluid flow also notably increases by 10-fold or more during inflammation [19,20,21,22]. However, it is currently unclear how much of the increased fluid flow is driven by elevated LN interstitial pressure compared to effects in peripheral tissues. Disruption to the integrity of fibroblast reticular cells (FRCs)-formed conduit network in the LN during inflammation correlated with a lack of FRC uptake of lymph-circulating dextran [23], suggesting that lymph flow within the LN parenchyma may be altered at sites of structural remodeling during LN swelling. However, it is also possible that the FRC extracellular matrix is required for dextran uptake while fluid flow remains unimpeded in other areas of the conduit system. Other studies have shown limited perturbation of the conduit network during the nodal inflammation induced by complete Freund’s adjuvant [24]. Further studies are therefore needed to correlate the effects of LN structural change on global lymph flow rates during standard and pathological inflammatory events.

After transport by lymphatic vessels, lymph fluid and its contents enter the first LN of a connected series of nodes. The series’ outermost LN is commonly called the draining or sentinel node (the latter being common in cancer-related and clinical studies). The chain of LNs provides sequential opportunities for pathogens traveling in lymph fluid to be removed from circulation. After passing through LNs, lymphatic vessels feed into the thoracic duct, which merges with the bloodstream. Therefore, pathogens not filtered by the LNs continue through lymphatic vessels to reach the blood [25,26].

Unlike blood, which remains relatively constant, the composition of lymph varies depending on location and physiology. Despite the filtering function of LNs, efferent (or post-nodal) lymph is enriched in total protein compared to incoming lymph. Lymph concentration occurs within the LN as water moves into the blood through LN blood capillaries and high endothelial venules (HEVs). Some proteins, such as immunoglobulins, are also enriched by their synthesis within and export from the LN. However, the LNs can also remove proteins from the lymph. The endogenous protein clearance capacity of LNs has been quantitated in rats, and many proteins were reduced in concentration in lymph after passage through the LN. This feature was independent of protein molecular weight [27].

The arrangement of LNs in tandem series allows the synergistic removal of pathogens or proteins on a larger scale than a single node could accomplish. Pathogen or dye uptake has been demonstrated to be serially smaller in LNs further downstream from the site of tissue entry [26,28]. Lymphatic drainage patterns from different tissues have been elucidated in mice using methods that inject lymph-circulating dye [29,30] or quantum dots [31,32,33], followed by visualization of LNs or lymphatic vessels [34,35]. One well-characterized lymph drainage route begins in the mouse’s hind footpad, draining to the popliteal LN (PLN) in the popliteal fossa (knee pit). From the PLN, lymph continues to the iliac and renal LNs. From the footpad, there is also a separate drainage route to the inguinal LN on the mouse’s flank [29]. From this less-efficient inguinal pathway, lymph drains to the axillary LN [29,36]. Other routes of drainage have also been defined in mice [30,33,34,37].

Humans have between 600 and 800 individual LNs [38]. Lymphatic drainage pathways in humans are more complex than in mice and show significant variation between individuals [39], as evidenced by studies tracking melanoma metastases [40,41]. In humans, the thoracic duct (the left lymphatic duct) empties lymph gathered from the legs, abdomen, and left side above the thorax into the left subclavian vein. The right lymphatic duct empties lymph from the right side of the body above the thorax into the right venous angle [42]. From the head, the left and right jugular lymphatic ducts empty into the left and right lymphatic ducts, respectively, at the root of the neck. Though relatively underexplored, differences exist in lymphatic transport from the left or right side, and drainage is not always symmetrical [40,43,44,45].

### 2.2. Lymph Nodes

LNs are secondary lymphoid organs with a highly organized and compartmentalized structure facilitating pathogen filtration, Ag acquisition, and lymphocyte activation [1]. LNs are unique among tissues because they receive input from two circulatory systems, and there is a requirement for larger biomolecules and free (non-cell associated) pathogens to enter through the lymph initially. Lymph enters the LN through the afferent lymphatic vessel (Figure 1). In most animals (one clear exception being swine), the afferent lymphatic vessel empties into a peripheral cavity separated from the node interior, known as a nodal sinus. Lymphatic sinuses are bordered by LECs, which form an essentially impermeable border to the LN interior [46]. Like blood endothelial cells (BECs) that prevent most large molecules from leaking out of the blood into the tissue, LECs prevent molecules larger than 70 kDa from passing through their border into the LN parenchyma. LEC-mediated size exclusion involves plasmalemma vesicle-associated protein 1 (PLVAP), which has been proposed to act as a molecular sieve [47,48]. The precise mechanisms through which PLVAP enforces size exclusion in the LN have not been determined.

Small molecular weight proteins that can pass through PLVAP-guarded LECs enter the conduit network of the LN. Conduits form a series of channels that connect the lymphatic sinuses to the HEVs located deeper in the LN parenchyma [49]. While conduits are formed by the stromal cells of the LN (specifically FRCs), dendritic cells (DCs) can also access small proteins moving through the conduits for Ag presentation [50]. We recently showed that viruses can also be transported in the conduit system to reach DCs in the LN interior [51].

The lymphatic sinus nearest the afferent lymphatic vessel is termed the subcapsular sinus (SCS) and is an essential site for Ag and pathogen capture (Figure 1). In naïve animals, the SCS is populated by sessile, tissue-resident CD169-expressing SCS macrophages (SSMs) and a smaller number of DCs [52]. SSMs can capture many incoming particulates from their location at the lymph/tissue interface, including viruses, bacteria, parasites, immune complexes, and even latex beads. Unlike robust partitioning in other anatomical sites of the LN, SSMs have somas (cell bodies) in the SCS and processes that extend through the sinus floor. SSMs can use their extended conformation to acquire Ag from the incoming lymph and relay this through the SCS floor into the LN parenchyma [53,54,55,56].

From the SCS, lymph flows around the periphery of the LN to transitional sinuses and then medullary sinuses (MS) that continue to filter the lymph [57]. This route of fluid movement is essential as infected SSMs can propagate and release some pathogens, which then flow to the MS. Like the SCS, the medullary sinus is populated by resident CD169^+^ medullary sinus macrophages (MSMs). MSMs can be distinguished from SSMs based on their anatomical location, morphology, cell surface markers (including F4/80 and CD64), and function, namely their comparatively higher phagocytic activity, lysozyme content, and lower pro-inflammatory cytokine expression [58,59,60,61]. MSMs likely act as a secondary filter for materials not fully captured by phagocytes in the SCS (or are propagated by SSMs) [62], as has been demonstrated for circulating Ag [63,64,65] and fluorescent dextran [66].

The central portion of LNs contains specialized sites for activating adaptive lymphocytes. Incoming naïve B and T cells enter the LN by cellular extravasation through specialized regions of blood vessels, the HEVs. Lymphocyte positioning after LN entry is determined by the localized expression and scavenging of multiple chemokines, including CCL19, CCL21, ACKR1, and CXCL13 [67,68,69,70,71], as well as other chemotactic molecules (such as sphingosine-1-phosphate and 25-dihydroxycholesterol). Most naïve B cells migrate into B cell follicles underneath the SCS after LN entry. A specialized subset of stromal cells, follicular dendritic cells (FDCs), also resides within follicles and is involved in follicular Ag capture and retention [54,60,72,73,74,75]. Notably, FDCs can retain intact Ags for weeks following exposure or inoculation [76], a feature recently shown to be facilitated by the low protease activity of the B cell follicle [77,78]. B cells can be activated extrafollicularly in the areas surrounding HEVs by Ag encounter before reaching the B cell follicle [74,79,80,81] and in medullary regions [82]. Activation of memory B cells has recently been shown to occur in foci near or in the SCS [83,84].

Naive T cells can be activated at different locations in the node, depending on the T cell subset and the source of Ag. Strategically positioned DCs can acquire Ag or pathogens near the LN sinus, activating T cells at the periphery of the node [85,86,87]. Migratory DCs arriving at the LN through the afferent lymphatics traverse the LECs of the sinus floor to migrate deeper within the T cell zone [88]. Migratory DCs can transfer Ag to tissue-resident XCR1^+^ DCs [89] that reside near HEVs [90]. As T cells enter the LN, they scan DCs for cognate Ag and arrest upon recognition [91,92,93]. T cell activation varies markedly depending on the pathogen, and activation against some pathogens relies almost exclusively on migratory DCs [94]. With other viruses, such as the vaccinia virus (VACV), priming can also occur via direct infection of APCs near the periphery of the LN [85,95]. For a review of T cell activation in the LN, please see the following [96,97,98].

Individual LNs are exposed to a unique assortment of Ags dictated by the peripheral tissues that they drain. Recent evidence suggests that the inflammatory-tolerance axis and the activated lymphocyte profiles may differ between distinct LNs [99]. Mesenteric lymph nodes (MLNs) drain the gastrointestinal tract, a frequent site of common viral infections in children. Additionally, MLNs are continuously exposed to foreign Ags from gut microbiota, and these nodes have a more tolerogenic profile than skin-draining LNs imparted by regulatory T cells (Tregs) [100,101] and tolerogenic DCs [102]. Notably, Tregs also populate the MLNs of germ-free mice, suggesting that these LNs may be predisposed to tolerance during early development [103]. Interestingly, MLNs are also a chain of multiple LNs draining unique sites in the gut, and tolerance induction is most robust in LNs draining the proximal small intestine [104]. Despite a propensity for tolerance, all MLNs can launch inflammatory responses to intestinal reoviral infection [105]. MLN stromal cells also induce gut-homing proteins (CCR9 and α4β7-integrin) in CD8^+^ T cells following oral.

Ag administration: this phenotype was not induced in vitro or in skin-draining LNs [106,107]. Interestingly, CD8^+^ T cells in mandibular LNs did not develop a gut-homing signature after oral infection by *Listeria monocytogenes* [108], suggesting this might be a specific feature of MLNs.

## 3. Lymphatic Viral Dissemination

### 3.1. Viral Movement to the LN After Initial Entry

Many human viruses disseminate within the host after initial replication in the LN, a route called lymphohematogenous dissemination. Viruses must first reach the LN to disseminate further through the efferent lymphatics. Viruses can arrive at LNs by infecting cells at the inoculation site or via drainage as free virions in the lymph. Viral replication and tissue inflammation induce the migration of infected or Ag-bearing DCs to the LN. The contribution of viral replication in tissue-resident cells resulting in viral release and drainage to the LN is an area of active investigation. Some well-described viral infections of specific LN cells are summarized in Table 1.

After inoculation into the tissue by needle, free infectious virus arrives at the LN within minutes [28,85]. When given by subcutaneous injection in the mouse footpad, VACV can be detected in the PLN within 3–5 min post-inoculation. Likewise, Zika virus (ZIKV) and adenovirus (AdV) have been shown to traffic rapidly to the PLN [28,85]. Viruses entering the LN through the lymph primarily encounter sinus-resident cells, including SSMs, MSMs, DCs, and LECs [28,51,85,109]. SSMs have now been shown to be infected by a wide array of viruses, including VACV [51,55,85,109,110], ZIKV [26,28,51], AdV [55], murine cytomegalovirus (MCMV) [111], murid herpesvirus-4 (MuHV-4) [112], West Nile virus (WNV) [113], vesicular stomatitis virus (VSV) [55,85,114], and Chikungunya virus (CHIKV) [115]. The exact receptors allowing SSMs to capture such a diversity of viruses are incompletely understood, and there is likely redundancy of non-specific receptors (such as scavenger receptors) and viral attachment factors. However, there is an indication that SSM infection may be receptor-dependent (rather than phagocytic), as SSMs poorly engulf virus-sized bare latex beads [55].

Infection of SSMs by lymph-borne virus results in a curious phenomenon that has yet to be fully explained: attrition of this vital layer of immune defense. Indeed, most viral infections lead to SSM attrition through viral cytotoxicity or some other stimulation, leaving the LN in a compromised state to respond to secondary infection [116,117]. Infected LN macrophages typically die within 24 h, although the cell death pathway responsible has been the subject of debate [116,118]. Macrophage attrition has also been demonstrated following vaccination with the inactivated influenza vaccine [119], raising the possibility that attrition may result from diverse inflammatory stimuli. A highly fulminant infection or a hefty dose of virus almost completely depletes LN sinuses of macrophages [28]. Depending on the extent of depletion, macrophages are restored from days to weeks through the recruitment of circulating monocytes and the ongoing proliferation of remaining SSMs [120]. The downstream consequences of macrophage attrition have yet to be entirely determined, although two models have been proposed. First, macrophage attrition may prevent continuous virus amplification in LNs [28]. Alternatively, attrition may enhance adaptive immune response initiation through unidentified signaling mechanisms or Ag distribution [26].

In addition to SSMs, other LN cells are susceptible to infection by lymph-borne viruses (Table 1). While fewer in number in LN sinuses than macrophages, sinus-associated DCs can capture viral Ags [82,86,87] and be directly infected [51]. Sinus-associated DCs have been proposed to sample particles more indiscriminately than LN macrophages, as SCS DCs can retain large fluorescent microspheres [87]. Certain viruses can also propagate inside B cell follicles, such as human immunodeficiency virus (HIV) (infecting CD4^+^ T follicular helper cells [121]), human herpesvirus 6 (HHV6, infecting CD4^+^ and CD8^+^ T cells [122,123]), and gammaherpesviruses (infecting B cells [124]). While SSM infection is thought to be partly non-specific by collecting viruses through phagocytosis, entry receptors have been well characterized for B and T cell infection (such as CD4 for HIV, CD46 for HHV6, and CD21 for Epstein–Barr virus). These viruses also establish latency within the LN by maintaining the viral genome in the nucleus or incorporating the viral genome into the host genome.

LECs can be infected by human cytomegalovirus (hCMV) [125] and arthritogenic alphaviruses [126]. These results enhance other recent findings of pro-inflammatory LEC functions, such as the potentiation of LN swelling, cytokine and chemokine expression, and Ag presentation [127]. There is also a burgeoning understanding of LEC functional specialization based on anatomical location in the node. Recent RNA sequencing data have classified LN LECs into at least five distinct subsets that correspond in humans and mice: valve LECs, SCS ceiling LECs, SCS floor LECs, MARCO^+^ MS LECs, and Ptx3^+^ MS LECs [127,128]. A single subtype of LECs has not been determined to be primarily responsible for viral sequestration and infection. Productive hCMV infection was observed in primary human LN LECs undistinguished by subtype [125], while MARCO^+^ LECs were found to sequester CHIKV particles [126]. As LEC subtypes have not yet been empirically distinguished from each other functionally, examining their respective susceptibility and contribution to lymphatic viral dissemination is an essential directive for future studies.

In addition to infecting LN cells as free virions in the lymph, viruses can also be carried into lymphatic vessels by migratory DCs that use these vessels as rapid-transit highways to the LN. Virological studies performed ex vivo have shown that myeloid cells with migratory potential, such as dermal Langerhans cells, can be infected by many different lytic viruses. However, for these viruses, little direct data exist conclusively demonstrating that infected cells can survive the trip from the tissue to the LN without succumbing to virus-induced cytolysis. Analysis of CHIKV infection revealed robust infection of inflammatory monocytes in the skin but little virus in monocytes in the draining LN [115]. Furthermore, some viruses impede peripheral myeloid cell migration [129] or cause peripheral lymphatic vessels to “zipper” shut [130]. Recently, Heim et al. demonstrated that memory T cells could exit the skin after VACV infection, trafficking to the draining LN via the lymphatics and seeding the LN with protective tissue-resident memory T cells [131]. Although this occurred after clearance of VACV-infected cells, it raises the possibility that other non-myeloid cells could potentially migrate to the LN via the lymphatics while carrying infectious virus.

To better understand lymphohematogenous dissemination, the Sigal Lab has extensively characterized LN infection with the mouse pathogen ectromelia virus (ECTV), a member of the orthopoxvirus genus. ECTV is experimentally administered to mice by footpad injection, where it replicates in the skin and disseminates through the lymphatics to the blood, eventually reaching and infecting hepatocytes [132]. ECTV also productively infects myeloid and B cells in the LN [133], making it a valuable model for investigating the factors that drive cell recruitment to infected LNs and antiviral protection within these nodes. Key determinants of ECTV-infected cell killing (thus limiting systemic spread) include LN-resident memory CD8^+^ T cells [134], CD4^+^ T cells [135], NK cells [136], inflammatory monocytes [133], and migratory DCs [137]. The TLR9-MyD88-IRF7 pathway is essential for migratory DCs (mDCs) in LNs to boost interferon-gamma (IFN-γ) expression by NK cells and type I innate lymphoid cells [138], NK cytolytic activity [136], and inflammatory monocyte recruitment [133]. Monocytes recruited to the draining LN respond to secreted IFN-γ by expressing CXCL9, amplifying NK recruitment [138]. ECTV-infected monocytes also produce type I interferon (IFN-α and IFN-β) after viral DNA is recognized by the signaling molecule STING [133]. These findings illustrate the multiple layers of communication within LNs, all poised to limit further viral dissemination.

The ability of viruses to access the lymphatics depends on many factors, including viral tropism, route of infection, and the anatomy of different peripheral tissues and their associated lymphatics. Several studies with disparate viruses have also shown that antiviral responses in the LN are negatively impacted by age [139,140,141]. Furthermore, an important emerging concept is that LNs are heterogeneous in function [142]. Likewise, specific lymph vessels differ depending on the tissue they perfuse. For instance, pulmonary lymphatic vessels lack the smooth muscle cells seen in most other lymphatic pathways [143], with respiratory movements thought to augment lung lymph circulation [144]. Lymph is also uniquely generated in the liver by highly fenestrated sinusoidal LECs [145], resulting in more highly concentrated lymph (with increased protein concentration) than in other tissues [144]. While comparative data are not available, the effect of different routes of pathogen entry into the lymphatics should be considered.

### 3.2. Lymphatic Contribution to Viral Spread

Ultimately, LNs must work together to capture all infectious viruses and prevent them from moving through the lymphatic network into the blood. Robust viral replication in the LN can overwhelm the filtering capacity of downstream nodes, allowing viruses to reach the blood via lymph transport [28,111,112,132,146,147,148]. Additionally, infected cells can carry viruses out of the LN. Many immune cells have been implicated in this process, including monocytes [149], neutrophils [150,151,152,153], and lymphocytes. In some cases, viral dissemination mediated by migratory cells such as monocytes likely plays a more critical role slightly later in infection. For example, when Zika virus infection is primarily restricted to LN and splenic macrophages, the virus reaches the blood but is not sustained for prolonged periods, suggesting another cell is responsible for amplification after LN infection [28].

Interestingly, LN macrophages can also sequester viruses to prevent an unusual, non-lymphatic vessel-mediated route of virus escape from the node: through LN nerves [114]. LNs are populated by unique sensory and sympathetic neurons that can modulate LN function [154]. These nerves preferentially innervate the periphery of the LN, where they can contact LECs and SSMs. When mice were infected with VSV in the footpad under conditions lacking SSMs, the virus escaped the LN through the nerve (instead of via the lymph or blood) [114]. These mice developed ascending paralysis and succumbed to CNS infection. This route has not been explored with other neurotropic viruses, including flaviviruses.

Some viruses establish chronic viral reservoirs within LN, mainly through the infection of cells in relatively privileged locations, such as the B cell follicle. Human immunodeficiency virus (HIV) is the most well-studied example, with HIV infecting and actively replicating in CD4^+^ T follicular helper cells [155]. Because most cytotoxic T cells do not express CXCR5, they do not readily enter B cell follicles to eliminate HIV-infected cells [156]. Additionally, FDCs can capture and retain HIV-Ab complexes through Fc receptor interactions, even when the virus is undetectable in the blood during the latent phase of the disease and antiretroviral treatment [155,157,158,159,160,161,162]. ZIKV, a positive-sense RNA virus without a latent cycle, can persist in B cell follicles for long periods [163,164]. The recent increase in viral genomic surveillance has suggested that additional viruses may also establish long-lived infections inside LNs [165,166,167].

## 4. Lymph Node Protection

### 4.1. Activation of LN B Cells by SSMs

While SCS macrophages remove infectious virus from the lymph, they also relay viral Ags to B cells and are particularly important for their activation [168]. Attenuation of B cell activation results from disruption of the SCS SSM layer by an earlier infection [116] or by administration of the TLR9 agonist CpG [116] or clodronate liposomes (CLLs) [55,169]. Despite this, SSM infection likely aids B cell activation in additional ways beyond Ag relay alone. Even after systemic ZIKV dissemination, maximal germinal center (GC) development relies on CD169^+^ macrophage infection [26]. However, despite accumulating evidence of the importance of SCS macrophages for nodal B cell responses, SSM depletion does not appear to reduce systemic levels of neutralizing Abs consistently [114,170].

Intravital microscopy has been used to visualize the acquisition of viruses by SSMs and the relay of viral Ag to underlying B cells. Cognate B cells migrate near the SCS and can be observed acquiring viral Ag [55]. Once activated in the LN by viral Ags, follicular B cells divide and form GCs that can be observed as soon as 5 days post-Ag uptake [171]. GC B cells undergo somatic hypermutation and affinity selection cycles that occur independently within each GC, maintaining a diverse range of epitopes recognized by the B cells within GCs and across the LN [172]. In response to viral infection, GCs may remain for months [173]. GC B cells eventually differentiate into antibody-producing plasma cells [174] or memory B cells [175]. Plasma cells can reside in LN medullary sinuses, but many long-lived plasma cells migrate to the bone marrow [172]. Memory B cells can also migrate to peripheral tissues or stay in the LN, particularly near the SCS [176,177].

### 4.2. T Cell-Mediated Protection Against LN Viral Infection

LN macrophages likely have a more negligible effect on antiviral T cell priming than other cells like DCs [178]. However, macrophages still modulate T cell activation by expressing modulatory cytokines, such as type I and II interferons, and activating other local phagocytes [95]. Nonetheless, antiviral T cells are activated in the LN during viral infection, where they can eliminate virus-infected cells before peripheral migration. In a series of elegant experiments, Halle et al. visualized CD8^+^ T cell-mediated killing of MCMV- and modified vaccinia Ankara virus (MVA)-infected cells in the LN. In this study, the efficiency of killing by OT-I CD8^+^ T cells (recognizing the MHC-class I molecule K^b^ in conjunction with the peptide SIINFEKL from ovalbumin [179]) was quantified [180]. As expected, CD8^+^ T cell killing was dependent on MHC-I expression by infected cells (as demonstrated by comparing WT MCMV with MCMV deficient in two viral regulators of Ag presentation). Surprisingly, the authors found that CD8^+^ T cells were not highly efficient in killing LN-infected cells, with only an average of 4.8 infected cells killed per day per T cell (on the lower end of values revealed through mathematical modeling [181,182]). These observations, combined with persistent CD8^+^ T cell motility only interrupted by brief contacts with infected cells, suggest that CD8^+^ T cells in LNs may prioritize continued surveillance over stringent cell killing. This hypothesis is strengthened by the literature showing that CD8^+^ T cell interaction with Tregs in LNs decreases their cytolytic activity ex vivo [183] and in vivo in LNs [184]. CD8^+^ T cells can also kill Ag-presenting LN DCs [185], suggesting that CD8^+^ T cells may modulate immune tolerance through APC pruning within the LN.

### 4.3. Cytokine Production in Response to LN Infection

Cytokines are the immune system’s messengers—they serve diverse functions in activating or otherwise regulating immune cells, and many have been implicated in preventing pathogen replication or spread. Accordingly, many cytokines have been studied for their contribution to controlling lymph-borne and LN-replicating viruses. One notable example is the type 1 interferon pathway (IFN-I). IFN-Is are expressed quickly after pathogen exposure and have myriad antiviral activities [186]. In the LN, IFN-I can activate DCs, potentiating their Ag-presentation and costimulatory function [187,188]. IFN-Is affect T cell activity in several ways, including influencing the expansion and effector differentiation of naïve T cells [189,190,191,192], central T memory cell localization to sites of LN infection [193], and exhaustion and death in activated T cells [194,195]. IFN-I also impacts the function of LN structural cells, including LECs and stromal cells, facilitating nodal expansion during the response to infection [17,196,197]. Likewise, LECs and stromal cells express inflammatory cytokines [198] regulating lymphocyte activation [195,199] and may even prevent viral dissemination from peripheral tissues [200].

In addition to sequestering viruses and activating B cells, SSMs also produce cytokines. Many studies have revealed that LN SCS macrophages are a potent source of IFN-I. IFN-I signaling is likely a key factor in preventing productive macrophage infection in the LN [60,114,178,201]. One of the defining cellular markers of SSMs and MSMs, CD169, encoded by the gene *Siglec1*, was suggested to be co-opted by HIV-1 to attenuate IFN-I signaling [202].

Recently, several studies have examined the production of IFN-Is and other antiviral cytokines by inflammatory monocytes within LNs after infection by ECTV [133], *Toxoplasmosis gondii* [203], VSV [204], lymphocytic choriomeningitis virus (LCMV) [204], and CHIKV [205]. Notably, an emerging theme from many of these studies has been the suppressive role of inflammatory monocytes in the LN, particularly regarding developing antiviral B cell responses. Adoptive transfer of VSV- and LCMV-specific B cells resulted in B cell apoptosis after interaction with inflammatory monocytes in infected LNs, facilitated by monocyte production of nitric oxide [204]. Similarly, infecting C57BL/6 mice with pathogenic CHIKV resulted in recruitment of neutrophils and inflammatory monocytes to the subcapsular and medullary sinuses, which inhibited lymphocyte recruitment and GC formation, and neutralizing Ab responses downstream of interferon-α/β receptor (IFNAR)-dependent nitric oxide production [205]. Whether LN-recruited inflammatory monocytes delay Ab response development rather than blunt them entirely remains to be seen. Consequently, further study is also needed to determine the degree to which inflammatory monocyte inhibition of B cells results from viral pathology versus a yet undescribed tolerance mechanism in LNs.

Interestingly, although plasmacytoid dendritic cells (pDCs) are characterized by the copious production of IFN-I [206,207], their role in IFN-I production in the LN during viral infection is mixed [208]. pDCs were dispensable for IFN-I expression after subcutaneous delivery of MVA [187]. However, pDCs were needed to develop maximal MVA-specific CD8^+^ T cell responses, primarily by recruiting the XCR1^+^ DCs required for T cell priming. pDCs in draining LNs were also shown to produce IFN-I during skin infection with HSV-1 [209].

Several other cytokines have also been implicated in the control of lymphatic-disseminating viruses. Serum levels of TNF-α, IL-6, and IL-1β increased after footpad injection of LPS, and exogenous delivery of these cytokines decreased lymphatic pulse rate and increased lymphatic vessel swelling [210]. TNF-α and IL-6 could also initiate a “shutdown” phase in sheep LNs [211], limiting lymphocyte egress from reactive LNs [212,213].

Intriguingly, naive PLNs transplanted into the mesentery of a chronic colitis mouse model expressed IL-4 as an anti-inflammatory inhibitor of T cell differentiation and disease pathogenesis [214], suggesting that chronic inflammation (such as in chronic infection) may alter the functional environment of the LN. LNs undergo significant structural changes during inflammation that are mediated by LN stromal cell expansion and remodeling [23,215], which express many types of cytokines. LN fibrosis that persists through antiretroviral therapy has also been observed during HIV infection [216]. These findings illustrate the contribution of immune and structural LN cells to maintaining the dynamic cytokine environment within LNs.

Chemokines are also robustly expressed in LN DCs [217,218], stromal cells [102,219], FRCs [220,221], and LECs [222,223,224] to recruit naïve lymphocytes into the node [225]. A recent paper from the Cyster lab [226] addressed the ongoing question of how lymphocyte recruitment into draining LNs is sustained during viral infection. Paradoxically, CCL21 is downregulated in the LN during infection and inflammation, yet this chemokine is considered essential for lymphocyte entry through HEVs. The authors found that two alternative and additive chemoattractant pathways compensated for decreased CCL21 expression: increased CCL19 expression in LNs (the other ligand for the CCR7 chemokine receptor, which is dispensable when CCL21 is present) and increased expression of the oxysterol enzyme Ch25 h in LN. The latter oxysterol pathway was also necessary for B cell recruitment to solid tumors [226], implicating its potential role as a general lymphocyte chemoattractant when CCL21 is downregulated (which also can occur during cancer).

### 4.4. Cooperation Between Recruited Innate Immune Cells

In addition to resident LN macrophages, migratory immune cells recruited into infected LNs play critical roles in controlling viral infection and aiding in B and T cell activation. Many LN cellular collaborations have recently been revealed between DCs, NK cells, and monocytes [227]. DC migration to draining LNs, which occurs following DC activation in peripheral tissues [228], recruits NK cells to LNs through IFN-γ production [229]. NK cells are present in low numbers in naïve LNs and are closely associated with medullary sinus DCs [230]. LN NK cells have a distinct molecular profile compared to blood-circulating NKs, including the upregulation of cytolytic molecules and lack of CCR7 expression [231], which may encourage them to remain in LNs. Activated NK cells produce IFN-γ [138], which upregulates their cytolytic activity [136] and recruits circulating monocytes into the LN [133]. Recruited monocytes, in turn, express CXCL9 to amplify NK cell recruitment further [138]. Activated NK cells migrate to the LN paracortex to aid in CD4^+^ T cell activation [230,232]. NK cells may also be vital for controlling chronic viral infections, as they have been shown to significantly reduce simian immunodeficiency virus (SIV) replication in African green monkey LNs [233]. In mouse models, chronic LCMV (clone 13) infection enhanced NK cell numbers compared to infection with an acute strain [234].

Finally, in addition to their suppressive roles in the LN during viral infection, monocytes have been shown to have several supportive functions. Monocytes colocalize with resident DCs in T cell zones to optimize T cell priming [235]. They also reduce lymphocyte egress through high expression of the chemoattractant sphingosine-1-phosphate (S1P) [236]. Monocytes can also differentiate into other myeloid cells, such as macrophages [120] and DCs [237]. Additionally, under specific circumstances, monocytes can directly present Ag to CD8^+^ (and CD4^+^ to a lesser degree) T cells [238].

### 4.5. Harnessing Protection in the LN Through Vaccination

**Figure 2:**
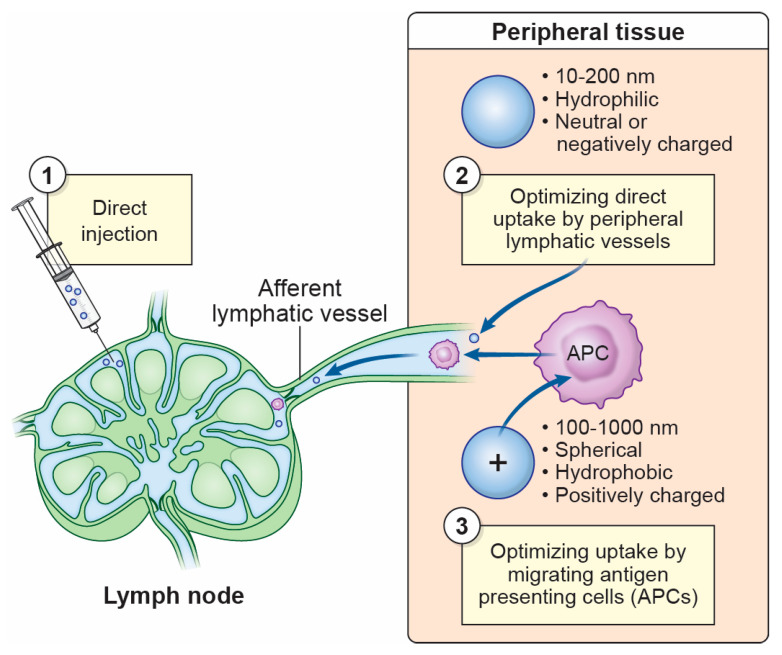
Strategies to maximize delivery of lymph-borne vaccines and therapeutics. (**1**) Antigen, vaccines, or antigen-presenting cells can be directly injected into a lymph node for enhanced antigen delivery and adaptive immune response development. (**2**) When administered in peripheral tissues, antigens and vaccines can be optimized for entry into lymphatic vessels with particle sizes of 10–200 nm, hydrophilicity, and a neutral or negative charge. (**3**) Antigens and vaccines can be optimized for antigen-presenting cell (APC) capture in peripheral tissues with particle sizes of 100–1000 nm, spherical shapes, hydrophobicity, and a positive charge. Vaccine-bearing APCs can migrate to draining lymph nodes for antigen relay and presentation.

Like the initiation of adaptive immune responses during LN viral infection, vaccine-produced Ags generally must be routed to the LN to activate B and T cells and develop protective memory. Therefore, an increased emphasis has been placed on optimizing Ag delivery via the lymphatics for presentation to the nodal B and T cells (Figure 2). Just as vaccine Ags have been optimized through an increased understanding of viral Ag presentation, much can be learned about the lymphatic delivery of viruses through our knowledge of vaccine movement to the LN.

The most direct approach to delivering vaccines to the LN has been through injection into the LN itself, sometimes in combination with adjuvants or chemokines. In preclinical and clinical studies, intranodal injection induced cellular and adaptive immune responses sufficient for disease treatment and, in some cases, was found to boost immunity compared to standard peripheral delivery routes [239,240,241,242]. Delivery of Ag-primed DCs for cancer immunotherapy by intranodal injection has also elicited an adaptive immune response [243,244,245]. Limitations to this approach include the invasiveness and technical difficulty of the procedure, challenges in locating specific LNs in more complex species (including humans), and a still-limited consensus on its efficacy across disease types [246]. While these drawbacks may limit the speed and scalability of intranodal injections needed for public vaccination programs, they may have specific uses, such as in cancer where intensive treatment and imaging are already occurring or in conditions that impede lymphatic drainage [246].

Efforts are also being made to improve the drainage of vaccines via the lymphatics when delivered into the muscle or skin. Biochemical alterations to vaccine carriers have been shown to significantly impact lymphatic uptake efficiency, with implications for both viral vector and nanocarrier vaccines. Particle size appears to be a major contributor to the ability of a vaccine to enter lymphatic vessels. While small particles can be rapidly cleared from the tissue by afferent lymphatic vessels (25 nm [247]) or leakage into blood vessels (<10 nm [248,249]), large particles (over 100 nm [247,250,251]) tend to remain at the delivery site. While the optimal size for lymphatic entry varies with the material used, sizes tend to fall within a range of 10–200 nm [246,247,252,253,254,255,256,257,258,259], similar to the size of virus particles (20–400 nm) [260]. Hydrophilic particles have also been shown to have higher entry into the lymphatics due to enhanced navigation of the hydrophilic interstitial matrix [259,261]. Likewise, positively charged carriers have a higher tendency to remain within the interstitium due to high concentrations of negatively charged hyaluronic acid on the extracellular matrix [255,262], implicating neutrally or negatively charged particles as also being more optimal for lymphatic transport [255,259,261,263]. Finally, the delivery route may significantly affect lymphatic Ag transport, with intradermal injection and microneedle delivery being ongoing candidates for vaccine delivery due to the unusually high oncotic pressure driving vaccines into the lymphatic pathway [264].

In addition to the delivery of free, non-cell-associated vaccines to the LN, APCs at the site of vaccine delivery have also been targeted to improve vaccine Ag delivery. The optimal conditions for DC Ag uptake and migration to the LN notably oppose those ideal for lymphatic vessel entry. DCs favor large, hydrophobic, and positively charged particles [261,265,266,267,268]. These preferences may result from APCs acquiring Ags through endocytosis, with hydrophobic and positively charged particles thought to interact with hydrophobic and negatively charged cell membranes [265,266,267,268]. DCs also preferentially acquire manufactured vaccine particles that approximate virus particles [269], with enhanced efficiency observed with spherical particles [259,270,271,272,273,274]. Interestingly, DCs can acquire and deliver Ags of up to 1000 nm to the LN [275,276]. While these opposing conditions optimal for DC and lymphatic-mediated Ag delivery seemingly pose a dilemma for optimal vaccine design, they ensure that Ag can reach the LN via one strategy or another. Ongoing vaccine studies often consider the effects of carrier design on delivery efficiency to LNs and the impact on subsequent immune responses.

Another consideration for vaccine effectiveness is the length of Ag delivery. Viruses that replicate in the tissue (especially at low levels) may drain to the LN for days or weeks. Recently, two papers revealed that “slow” vaccine delivery to the LN enhances the development of humoral immune responses. First, subcutaneous osmotic pump implantation modeled slow vaccine delivery in rhesus macaques, allowing continuous vaccine release for up to 4 weeks. Alternatively, the authors used increasing vaccine doses in multiple injections over two weeks [277]. Both slow delivery methods resulted in more potent and diverse B cell responses and higher Ag retention in draining LNs [277,278]. Later, dose escalation in rhesus macaques was shown to elicit GCs lasting up to 6 months [279]. These delivery protocols may enhance humoral responses by ensuring continual Ag exposure in draining LNs, more closely modeling Ag exposure during pathogen replication. Future studies could combine adjuvant and slow delivery, perhaps providing a promising regimen for challenging vaccination scenarios.

Although viruses that disseminate into the blood can be distributed throughout the body, vaccines only reach the draining LN. Recently, interest has sparked during human vaccination regarding the impact of the site of prime-boost vaccine regimens (e.g., the prime and boost doses are administered in opposite arms). Results addressing this question have so far been mixed. Ipsilateral boosting has been associated with more significant B cell clonal expansion and Ag cross-reactivity in primed LNs [280,281,282], suggesting that a portion of primed B cells remain in LNs for recall for the duration of a typical prime-boost regimen. However, ipsilateral (same side as the injection) and contralateral (opposite side) delivery routes have also been shown to elicit similar immunity in a clinical study on infants receiving the DTaP-IPV-Hib-Heb vaccine [283]. Similar immunity and protection were also observed during SARS-CoV2-mRNA vaccination in mice [284]. Differences in dosage, vaccine platform, and the timing of sample collection potentially contribute to the different results of these studies.

Many of the same considerations for vaccine carrier design also extend to therapeutic drug carrier design for lymphatic transport. Since lymphatic vessel-targeted drugs would ideally have limited sequestration by peripheral tissue cells, their design has primarily focused on direct entry into lymphatic vessels. For instance, the hydrophilicity of drug delivery vectors has been directly modified through the introduction of polyethylene glycol groups (PEGylation) [263,285,286,287] and the addition of sianol groups (Si-OH) [288] to enhance lymphatic uptake [259]. A specific therapeutic targeting method that has shown promise in preclinical testing is the use of superparamagnetic iron oxide nanoparticles (SPIONs), which can be induced to accumulate in an LN of interest with an applied magnetic field [289,290,291,292]. The intended effects of lymphatic targeting drugs include stimulating B and T cells with lymphatically delivered cytokines, eliminating LN-resident or migrating tumor cells, and eliminating latent LN viral reservoirs such as HIV [293,294]. Therapeutic lymphatic targeting is also being explored for imaging purposes, with intranodal injection of gadolinium-labeled nanoparticles [295] or peripheral injection of SPIONs [291,292] providing high specificity for magnetic resonance imaging.

## 5. Conclusions

The lymphatic system dictates the balance between viral control and dissemination. Our knowledge of the lymphatic organization and function has rapidly advanced in recent years due to increasing interest and general technological advances. High-throughput transcriptional sequencing, advanced imaging techniques such as intravital microscopy, and the development of many viral genetic systems for virus visualization and functional modulation have all been applied to the study of viral infection of the lymphatics. Unanswered basic scientific questions in this field reflect the complexity and heterogeneity inherent to specific pathogens and individuals. In addition to expanding the breadth of viruses used for investigating lymphatic infection and dissemination, future studies must identify unifying principles that address but do not oversimplify the diversity of findings from unique pathogens and tissues. As more becomes known, our ability to harness the full power of the LN through enhanced delivery of therapeutics and vaccines creates boundless opportunities to understand this complex system better. The continued emergence of viruses with community spread potential and the need for improved therapeutics against established pathogens underscores the importance of a better understanding of all routes of viral spread, including through lymphatics.

## Figures and Tables

**Table 1:** Summary of viruses known to infect LN cells.

Virus	LN Cell Type	Productive Infection?
Adenovirus	SSMs	Yes
Chikungunya virus	SSMs, LECs	Yes (SSMs), unknown (LECs)
Ectromelia virus	B cells, myeloid cells	Yes
Gammaherpes viruses	B cells	Yes
Human Cytomegalovirus	LECs	Yes
Human herpesvirus 6	CD4 T cells	Yes
Human Immunodeficiency virus	CD4 T cells	Yes
Murine Cytomegalovirus	SSMs	Yes
Murid herpesvirus-4	SSMs	Yes
West Nile virus	SSMs	Yes
Zika Virus	SSMs	Yes

## Data Availability

No new data were created or analyzed in this study.

## Abbreviations

The following abbreviations are used in this manuscript:

Ab	Antibody
Ag	Antigen
APC	Antigen presenting cell
AdV	Adenovirus
BEC	Blood endothelial cell
CHIKV	Chikungunya virus
CLL	Clodronate liposome
CNS	Central nervous system
DC	Dendritic cell
ECTV	Ectromelia virus
FDC	Follicular dendritic cell
FRC	Fibroblast reticular cell
GC	Germinal center
HCMV	Human cytomegalovirus
HEV	High endothelial venule
HIV	Human immunodeficiency virus
IFN-g	Interferon gamma
IFN-I	Type I interferon
IFNAR	Interferon-a/b receptor
LCMV	Lymphocytic choriomeningitis virus
LEC	Lymphatic endothelial cell
LN	Lymph node
MCMV	Murine cytomegalovirus
mDC	Migratory dendritic cell
MS	Medullary sinus
MSM	Medullary sinus macrophage
MuHV-4	Murid herpesvirus-4
MVA	Modified vaccinia Ankara virus
NK cell	Natural killer cell
pDC	Plasmacytoid dendritic cell
PEG	Polyethylene glycol
PLN	Popliteal lymph node
PLVAP	Plasmalemma vesicle associated protein 1
S1P	Spingosine-1-phosphate
SARS-CoV2	Severe acute respiratory syndrome coronavirus 2
SCS	Subcapsular sinus
Si-OH	Sianol
SPION	Superparamagnetic iron oxide nanoparticle
SSM	SCS macrophage
VACV	Vaccinia virus
VSV	Vesicular stomatitis virus
WNV	West Nile virus
ZIKV	Zika virus
